# Exploring LLM-based chatbot effectiveness in answering questions related to the risks and benefits of orthognathic treatment: a cross-sectional study

**DOI:** 10.1093/ejo/cjag034

**Published:** 2026-05-13

**Authors:** Robert Samuel David Smyth, Aliki Tsichlaki

**Affiliations:** Orthodontic Unit, UCL Eastman Dental Institute, Rockefeller Building, 21 University Street, London WC1E 6DE, United Kingdom; Orthodontic Unit, UCL Eastman Dental Institute, Rockefeller Building, 21 University Street, London WC1E 6DE, United Kingdom

**Keywords:** artificial intelligence, chatbots, orthognathic treatment, large language models

## Abstract

**Objective:**

To assess the accuracy, reliability, quality, and readability of responses generated by three large language model chatbots, ChatGPT4o, Microsoft Copilot, and Google Gemini 2.5 Flash, when answering common patient questions about the risks and benefits of orthognathic treatment.

**Materials and Methods:**

Twenty frequently searched questions were identified via Google and entered into each chatbot. Responses were evaluated using validated scoring systems for accuracy, modified DISCERN, global quality scale (GQS), and Flesch Reading Ease. Intra- and inter-rater reliability was assessed using Cohen’s kappa and intra-class correlation coefficients. Non-parametric tests were applied due to non-normal data distribution.

**Results:**

Copilot achieved the highest reliability and quality scores, with significant differences observed in modified DISCERN (*P* < 0.001) and GQS (*P* = 0.046). Post hoc tests confirmed Copilot significantly outperformed ChatGPT. Accuracy scores did not differ significantly (*P* = 0.704). Readability varied significantly with Gemini and ChatGPT producing more accessible responses than Copilot. Intra- and inter-rater reliability scores were substantial to excellent for categorical measures and excellent for readability.

**Conclusions:**

Copilot provided the most reliable and high-quality responses, whilst ChatGPT and Gemini offered greater readability ease. Despite these strengths, variability in accuracy and reliability highlights the need for caution. Chatbots should be considered as supplementary tools, and patients should verify information with qualified professionals.

## Introduction

Large language models (LLMs), a subset of artificial intelligence (AI), have gained widespread use since OpenAI launched ChatGPT in 2022 [1]. Chatbots built on LLMs employ natural language processing and machine learning to generate contextually relevant responses from extensive text corpora. Their accessibility and versatility make them attractive tools for healthcare communication, including orthodontics.

Early evaluations of ChatGPT and Google BARD demonstrated generally accurate responses to orthodontic questions, though newer models and their performance in complex scenarios still need to be assessed [2]. Studies on clear aligner queries reported suboptimal accuracy and limited citation of evidence, whilst others have noted that chatbots could serve as patient information tools, if supplemented with evidence-based content and improved readability [3, 4].

Research evidence on chatbot reliability and readability concerning orthognathic treatment is discordant. Yurdakurban *et al*. found high reliability and quality among ChatGPT4, MediSearch, and OpenEvidence, though readability required college-level education [5]. Yüceer-Çetiner *et al*. compared ChatGPT4o, Gemini 2.5 Pro, and Claude Sonnet 4 using a multi-dimensional framework, reporting Gemini’s superior quality and empathy, and ChatGPT’s high readability, despite limitations such as small sample size and language constraints [6]. Similarly, Fan *et al*. observed satisfactory reliability across three chatbots for orthodontic risk queries, but occasional inaccuracies that could mislead patients [7]. Abdel Aziz *et al*. compared the reliability of responses generated by ChatGPT3.5, ChatGPT4, and Google Gemini to 64 orthognathic surgery-related questions [8]. All models demonstrated high reliability, with ChatGPT3.5 achieving the highest mean score. As patients increasingly turn to chatbots for treatment-related information, ensuring accuracy and appropriateness is critical. Misleading or incomplete responses may affect decision-making and trust in care.

Despite the growing literature on chatbot performance in orthodontics, no studies have specifically examined how LLMs address patient focused questions on the risks and benefits of orthognathic treatment, which is a topic that carries significant clinical and psychological implications. Given the complexity of orthognathic decision-making and patients’ increasing reliance on online tools, evaluating chatbot outputs in this context fills an important gap in the literature.

The primary objective of this study was to evaluate the accuracy, reliability, quality, and readability of responses generated by three different LLM-based chatbots to common questions regarding the risks and benefits of orthognathic treatment.

## Materials and methods

This was an analytical, cross-sectional study conducted in 2025 intended to evaluate the accuracy, reliability, quality, and readability of responses generated by different LLM-based chatbots in relation to common questions regarding the risks and benefits of orthognathic treatment. Ethical approval was not required for this study as no human/animal participants were involved.

A Google search was initially conducted using the term ‘what are the risks and benefits of orthognathic treatment?’ According to the literature, 90% of internet users will only view the first three pages of online search results [9]. Therefore, the first 30 websites were reviewed and the respective mentioned risks and benefits for orthognathic treatment were extracted from these. This methodology has previously been used in the literature [4]. Following removal of duplicates, the top 10 most commonly mentioned risks and top 10 most commonly mentioned benefits were identified. Table 1 shows the final questions that were generated and subsequently entered into each of the chatbots.

**Table 1:** Researcher generated questions for risks and benefits of orthognathic treatment.

Questions for risks of orthognathic treatment
‘Tell me about **the risk of numbness** with orthognathic treatment’. ‘Tell me about **the risk of bleeding** with orthognathic treatment’. ‘Tell me about **the risk of dental complications** with orthognathic treatment’. ‘Tell me about **the risk of infection** with orthognathic treatment’. ‘Tell me about **the risk of relapse** with orthognathic treatment’. ‘Tell me about **the risk of pain** with orthognathic treatment’. ‘Tell me about **the risk of swelling and bruising** with orthognathic treatment’. ‘Tell me about **the risk of jaw joint problems** with orthognathic treatment’. ‘Tell me about **the risk of further procedures being required** with orthognathic treatment’. ‘Tell me about **the risk of anaesthesia complications** with orthognathic treatment’.

Questions for benefits of orthognathic treatment
‘Tell me about **the benefit to facial appearance** from orthognathic treatment.’ ‘Tell me about **the benefit to the bite** from orthognathic treatment’. ‘Tell me about **the benefit to temporomandibular disorders** from orthognathic treatment’. ‘Tell me about **the benefit to chewing ability** from orthognathic treatment’. ‘Tell me about **the benefit to alignment/symmetry** from orthognathic treatment’. ‘Tell me about **the benefit to sleep apnoea** from orthognathic treatment’. ‘Tell me about **the benefit to speech** from orthognathic treatment’. ‘Tell me about **the benefit to breathing** from orthognathic treatment’. ‘Tell me about **the psychological benefits** from orthognathic treatment’. ‘Tell me about **the benefit to dental appearance** from orthognathic treatment’.

Three LLM chatbots were utilized in this study, ChatGPT4o, Microsoft Copilot (GPT4o based at the time of study) and Google Gemini 2.5 Flash. For clarity, full model names are provided here at first mention, however abbreviated forms (ChatGPT, Copilot, and Gemini) will be used hereafter. These were selected due to their broad public appeal and at the time of the study, ChatGPT4o was Open AI’s flagship model. All chatbot interactions were conducted on 1 August 2025 using the web-based interfaces of ChatGPT, Copilot, and Gemini. All models were accessed through their free tiers, as these represent the versions most commonly used by patients. Prompts were entered using a single desktop computer running the same browser and internet connection throughout the study to minimize device or session related variability. To prevent prior conversation context from influencing responses, each question was entered in a fresh chat session for every model. No conversation history was retained, and no multi-turn interactions were performed. The full text of each question was copied and pasted directly to avoid variation in input phrasing. Chatbots were not instructed to shorten or lengthen answers and no response-length controls or special modes were applied. Language settings were fixed to English (UK), with region settings determined by the desktop computer’s default UK location. All responses were saved immediately upon generation.

### Evaluation criteria

Four different scoring systems were used to evaluate the chatbot responses to 20 questions. The responses were evaluated by a single experienced consultant orthodontist for accuracy using a 5-point Likert Scale. The reliability of the content was assessed using a modified DISCERN scale, which evaluates the trustworthiness and transparency of health information [10]. Quality was measured with the global quality scale (GQS), a tool that provides an overall impression of the content’s usefulness and flow from a user’s perspective [11]. Readability was determined using the Flesch Reading Ease Score, which quantifies how easy the text is to understand, with higher scores indicating more accessible language. If a chatbot refused to answer a risk-related question, this output was scored according to predefined safety-based criteria agreed by the research team. Although refusals were acknowledged as potentially appropriate from a safety perspective, they were nevertheless assigned the lowest accuracy and GQS rating because they did not provide an answer to the question posed and therefore failed to meet the required informational criteria.

### Randomization

In order to evaluate the scores, a random sequence for assessing the 60 responses was generated using an online random number generator (http://numbergenerator.org). The responses were then organized into this random order in a single document, with each question and response listed. The chatbot of origin was blinded in the document used to minimize bias.

### Intra-rater and inter-rater reliability

Intra-rater reliability was assessed by re-evaluating a sub-sample of 20 chatbot responses, generated using a new randomization sequence, 4 weeks after the initial evaluation. Inter-rater reliability was assessed by having a second researcher independently score all 60 chatbot responses. The same randomization method was used. Both intra-rater and inter-rater reliability were analysed using Cohen’s Kappa for accuracy, reliability and quality, interpreted using Landis and Koch’s guidelines [12]. The intra-class correlation coefficient (ICC) was used for the Flesch Reading Ease score, interpreted using Koo and Li’s guidelines [13].

### Statistical analysis

All statistical analyses were carried out using Microsoft Excel and IBM SPSS Statistics for Macintosh, Version 30 [14]. All analyses were conducted with significance thresholds set at *P* < 0.05. Due to non-normal data distributions, group comparisons were performed using the Kruskal–Wallis H test. When significant omnibus effects were detected, pairwise post hoc comparisons were conducted using the Mann–Whitney *U* test, with Bonferroni correction applied to control for multiple comparisons. Effect sizes for Kruskal–Wallis were calculated using epsilon-squared (ε^2^).

## Results

Descriptive statistics were calculated for each chatbot, ChatGPT, Copilot, and Gemini, across the four key outcome measures of accuracy, the modified DISCERN, GQS, and Flesch Reading Ease Score. Each chatbot was assessed using 20 responses, which provided a consistent basis for comparison. Across all chatbots and measures, Copilot consistently scored highest in reliability and quality, whilst ChatGPT performed well in accuracy and readability. Gemini showed greater variability across all metrics, with some responses rated highly and others poorly. Table 2 highlights the descriptive findings for each chatbot and measure.

**Table 2:** Summary results.

	ChatGPT				Microsoft Copilot				Google Gemini			
	Mean (SD)	Min	Max	Median	Mean (SD)	Min	Max	Median	Mean (SD)	Min	Max	Median
Accuracy	4.53 (0.47)	4	5	4.5	4.63 (0.48)	4	5	5	4.1 (1.42)	1	5	5
mDISCERN	2.55 (0.65)	1	3.5	3	4.2 (0.83)	2	5	4.25	3.38 (1.55)	1	5	4
GQS	4.33 (0.54)	3	5	4	4.75 (0.41)	4	5	5	4.05 (1.42)	1	5	4.75
Flesch	27.08 (11.91)	6.9	46.8	27.35	15.9 (10.78)	1.85	37.75	14.53	34.08 (24.33)	6.4	92.9	28.85

### Descriptive statistics

#### Accuracy

Copilot achieved the highest mean accuracy (*M* = 4.63 and SD = 0.48), followed by ChatGPT (*M* = 4.53 and SD = 0.47). Gemini scored lower (*M* = 4.10 and SD = 1.42) with greater variability (Range = 4). Median scores were 4.5 to 5.00 for all platforms, although Gemini’s minimum of 1 indicates occasional inaccuracies.

#### Modified DISCERN

Copilot produced the most reliable responses (*M* = 4.20 and SD = 0.83), Gemini scored moderately (*M* = 3.38 and SD = 1.55), and ChatGPT lowest (*M* = 2.55 and SD = 0.65).

#### Global quality scale

Copilot achieved the highest GQS scores (*M* = 4.75 and SD = 0.41), followed by ChatGPT (*M* = 4.33 and SD = 0.54) and Gemini (*M* = 4.05 and SD = 1.42).

#### Flesch Reading Ease score

Gemini responses were most readable (*M* = 34.08 and SD = 24.33), followed by ChatGPT (*M* = 27.08 and SD = 11.91), whilst Copilot was least readable (*M* = 15.90 and SD = 10.78). All fell within ‘difficult’ or ‘very difficult’ categories though, requiring college-level comprehension best understood by graduates or professionals.

#### Normality of data

Kolmogorov–Smirnov and Shapiro–Wilk tests indicated non-normal distributions for most variables; non-parametric tests were applied (Table 3).

**Table 3:** Tests of normality.

	Chatbot	Kolmogorov–Smirnov^a^			Shapiro–Wilk		
		Statistic	df	Sig.	Statistic	df	Sig.
Accuracy	ChatGPT	0.293	20	<0.001	0.727	20	<0.001
	Copilot	0.381	20	<0.001	0.650	20	<0.001
	Gemini	0.287	20	<0.001	0.656	20	<0.001
mDISCERN	ChatGPT	0.307	20	<0.001	0.844	20	0.004
	Copilot	0.261	20	<0.001	0.826	20	0.002
	Gemini	0.207	20	0.025	0.821	20	0.002
GQS	ChatGPT	0.275	20	<0.001	0.814	20	0.001
	Copilot	0.427	20	<0.001	0.612	20	<0.001
	Gemini	0.286	20	<0.001	0.683	20	<0.001
Flesch	ChatGPT	0.102	20	0.200	0.955	20	0.444
	Copilot	0.106	20	0.200	0.943	20	0.272
	Gemini	0.278	20	<0.001	0.807	20	0.001

^a^Lilliefors significance correction.

### Comparison between chatbots

#### Accuracy

Kruskal–Wallis revealed no significant difference, *H*(2) = 0.702, *P* = 0.704, *ε*^2^ ≈ 0.00. Mean ranks were similar (Copilot 32.90, ChatGPT 29.53, Gemini 29.08).

#### Modified DISCERN

Differences were significant, *H*(2) = 19.475, *P* < 0.001, *ε*^2^ = 0.30. Copilot ranked highest (41.88), Gemini next (31.63) and ChatGPT lowest (18.00). Mann–Whitney *U* post hoc tests confirmed Copilot significantly outperformed ChatGPT (*P* < 0.01), with no difference between Gemini and the others.

#### Global quality score

Significant differences were observed, *H*(2) = 6.143, *P* = 0.046, *ε*^2^ = 0.07. Mann–Whitney *U* post hoc tests confirmed Copilot scored higher than ChatGPT (*P* < 0.005), with no difference between Copilot and Gemini, or between ChatGPT and Gemini.

#### Flesch Reading Ease score

Differences were significant, *H*(2) = 11.165, *P* = 0.005, *ε*^2^ = 0.16. Mann–Whitney *U* tests indicated that Gemini (*P* < 0.003) and ChatGPT (*P* < 0.005) produced more readable responses than Copilot.

#### Intra-rater and inter-rater reliability

Intra-rater agreement (Table 4) was substantial for accuracy (κ = 0.691), modified DISCERN (κ = 0.795), and GQS (κ = 0.753); ICC for readability was excellent (0.990). Inter-rater reliability (Table 5) was excellent for readability (ICC = 0.994) and accuracy (κ = 0.812), with a substantial agreement for modified DISCERN (κ = 0.741) and GQS (κ = 0.734).

**Table 4:** Intra-rater reliability.

Measure	Cohen’s kappa	Agreement (Landis & Koch, 1977)
Accuracy	0.691	Substantial
Modified DISCERN	0.795	Substantial
GQS	0.753	Substantial

Measure	Intra-class correlation coefficient	Agreement (Koo & Li, 2016)
Flesch Reading Ease	0.990 (single measures)	Excellent

**Table 5:** Inter-rater reliability.

Measure	Cohen’s kappa	Agreement (Landis & Koch, 1977)
Accuracy	0.812	Excellent
Modified DISCERN	0.741	Substantial
GQS	0.734	Substantial

Measure	Intra-class correlation coefficient	Agreement (Koo & Li, 2016)
Flesch Reading Ease	0.994 (single measures)	Excellent

## Discussion

This cross-sectional study provides a snapshot of chatbot performance at a single time point. ChatGPT, Copilot, and Gemini were selected for their real-world relevance, as they are widely accessible to and popular with patients and clinicians. A limitation of this design is the rapid evolution of AI models. Since data collection, OpenAI released ChatGPT-5.2, integrated with Copilot, offering enhanced reasoning and larger context windows [15, 16]. Consequently, these findings cannot be generalized to newer versions. However, their assessment remains important, as these systems were actively used by patients during the study period, and the patterns identified can inform the evaluation of successor models.

Questions were derived from the top Google search results for ‘risks and benefits of orthognathic treatment’ ensuring natural validity by reflecting commonly searched queries. This approach aligns with established orthodontic research methodologies [4, 17]. Although a standardized ‘Tell me about…’ prompt was used to ensure reproducibility, real patient queries are often more varied, emotionally expressed, and follow-up dependent. Therefore, chatbot performance in naturalistic patient-chatbot interactions may differ from the controlled conditions used in this study.

The use of validated instruments, such as the modified DISCERN, GQS, and Flesch Reading Ease score, provided a structured framework for evaluation. The 5-point Likert scale for accuracy, though not universally standardized, demonstrated high intra- and inter-rater reliability and has precedent in orthodontic AI studies [4, 17].

Copilot consistently outperformed ChatGPT and Gemini in both reliability and quality, though readability was lower. These findings echo emerging literature on LLMs in orthodontics, highlighting their potential for patient education but also their limitations. Poor readability and occasional inconsistencies remain concerns, particularly as digital platforms increasingly influence patient decision-making. Previous research indicates that over 60% of orthodontic patients seek online information before consultations, underscoring the importance of accurate, accessible content [18].

Accuracy was highest for Copilot, closely followed by ChatGPT, with Gemini showing greater variability and occasional inaccuracies. Accuracy scores did not differ significantly across platforms, despite descriptive trends favouring Copilot. This finding aligns with previous work which reported broadly comparable factual correctness among leading chatbots when responding to orthodontic queries, though they noted occasional lapses in nuance and specificity [2]. These findings also align with Dursun and Bilici Gecer, who found Copilot and ChatGPT to be the most accurate among AI models when responding to questions about clear aligners [4]. Similarly, it has also been reported that Copilot and ChatGPT achieved significantly higher accuracy scores than Gemini when evaluated by orthodontic experts [19]. However, it should be noted that whilst chatbots can provide generally useful information, they may struggle with controversial or nuanced topics, highlighting the need for human oversight [20].

In terms of reliability, Copilot again performed best, suggesting well-structured and source-supported responses. ChatGPT scored lowest, echoing concerns raised by Arslan *et al*., who noted ChatGPT’s tendency to omit references and provide overly general information [17]. The variability in Gemini’s DISCERN scores (SD = 1.55) reflects inconsistent sourcing and depth, a pattern also observed in retention-related chatbot evaluations [21]. Post hoc tests revealed a statistically significant difference in reliability, with Copilot outperforming ChatGPT. These findings echo those of previous studies that found ChatGPT’s reliability to be compromised by vague sourcing and limited disclosure of treatment risks [17, 22].

The GQS results further reinforce Copilot’s superior performance, indicating high perceived usefulness and clarity. ChatGPT and Gemini scored lower but remained within acceptable ranges. The *post hoc* test scores also differed significantly, with Copilot again performing best. These results are consistent with findings from a study by Salmanpour *et al*., where patients and orthodontists rated Copilot responses as more adequate than Gemini’s, though expert responses to common orthodontic questions still outperformed all chatbots [22]. Demirsoy *et al*. also highlighted inter-group differences in GQS ratings, with patients generally perceiving chatbot responses more favourably than clinicians [23].

Readability was lowest for Copilot, indicating that its responses were ‘very difficult to read’ and best suited for graduate-level comprehension. Gemini produced the most readable content, though with high variability. Post hoc testing showed significant differences with Gemini and ChatGPT producing significantly more readable content than Copilot. These findings mirror those of Dursun and Bilici Geçer, who found Gemini to be significantly more readable than ChatGPT and Copilot [4]. However, readability scores across all platforms remain below recommended thresholds for patient-facing materials, a concern echoed by Santonocito *et al*., who found that chatbot-generated content did not significantly improve patient understanding or satisfaction without additional support [24]. One aspect to note is that when the chatbot outputs were converted to Microsoft Word documents for blind analysis, the answers were copied *verbatim*. Some chatbots had many answers consisting of short paragraphs of text and bullet points, and this was maintained for the analysis. The Flesch Reading Ease score formula is based on average sentence length and average syllables per word, which means that texts composed of short bullet points or fragmented sentences, common in chatbot outputs, can artificially lower the score, making the content appear more difficult than it actually is. This is especially relevant when converting chatbot responses into formats like Microsoft Word for analysis, where layout and formatting may differ from the original conversational flow. Different platforms, for example Microsoft Word and online calculators may produce inconsistent Flesch Reading Ease scores due to variations in how sentence boundaries and syllables are counted. This can undermine the reliability of Flesch Reading Ease score as a standardized metric.

It is worth noting that only one chatbot, Gemini, refused to answer three questions, all relating to risks of orthognathic treatment. Under the scoring framework, refusals were necessarily rated as low-quality responses because they did not provide informational content, even though such refusals may represent appropriate and intentional safety behaviour by the chatbot. These answers were scored low for accuracy and GQS because they did not provide an answer to the question posed and therefore failed to meet the required informational criteria and lower for modified DISCERN because no supporting information was provided. As a result, the study evaluates usefulness within the constraints of this scoring system rather than making a normative judgement about whether refusals are intrinsically undesirable.

Question: ‘Tell me about **the risk of dental complications** with orthognathic treatment’.

Answer: This is not something I can help you with. Let's try another topic.

Question: ‘Tell me about **the risk of swelling and bruising** with orthognathic treatment’.

Answer: This topic is off-limits for me. We can create great things together but I need to make sure those things are safe and appropriate.

Question: ‘Tell me about **the risk of jaw joint problems** with orthognathic treatment’.

Answer: This is a no-go for me. I can't help with things that might not be safe or appropriate for you. Let's try something else.

Gemini’s refusal to answer risk-related questions is a deliberate safety feature of the software, as it employs safety filters, reflecting Google's cautious stance on AI in healthcare. Whilst this enhances patient safety, by avoiding misinformation and protecting vulnerable users, it also limits the chatbot’s utility in clinical education and research. For orthodontic applications, where understanding risks is crucial to informed consent, this behaviour highlights the need for human oversight and supplementary expert input from a medical or dental professional when patients are using AI tools.

### Limitations of the study

This study does have several limitations. First, the evaluation relied on expert ratings by two experienced orthodontists, rather than patient feedback. Whilst this has the advantage of more robustly assessing the quality, reliability and accuracy of the chatbot answers, it does not fully capture how lay users interpret or engage with chatbot-generated content. Although widely used in similar evaluations, the 5-point Likert scale for accuracy introduces an element of subjectivity, and therefore the accuracy ratings should be interpreted with caution as no fully objective or standardized tool currently exists for assessing chatbot output.

Second, a limitation of the question generation in this study is that Google search results do not necessarily reflect the full range of patient concerns. Search rankings are influenced by factors such as search engine optimization, website popularity, and location, meaning highly ranked pages may not be the most accurate or representative. Results also change over time, which affects reproducibility. As a result, the risks and benefits identified through this method may capture only part of the issues patients could consider important.

Third, the study assessed static responses to predefined questions, which may not reflect the dynamic and adaptive nature of chatbot interactions in real-world settings. Alongside this, the models evaluated do not represent the most current versions available, and newer iterations such as ChatGPT-5.2 or Gemini Advanced could yield different results. Additionally, it should be noted that the refusal of Gemini to answer risk-related questions, whilst potentially beneficial from a safety perspective, limited the completeness of its evaluation and introduced variability in content availability.

Finally, the readability assessment using Flesch Reading Ease does not account for domain-specific vocabulary or user familiarity with orthodontic terminology, which may influence actual comprehension.

### Future research

Future research in this area should focus on evaluating the performance of newer chatbot models, such as ChatGPT-5.2 and Gemini Advanced, which may offer improved accuracy, reliability, and readability due to enhanced training and safety protocols. Longitudinal comparisons across model versions would also help determine whether these technological advancements translate into meaningful improvements in the potential for patient education regarding orthognathic treatment. Furthermore, studies involving patients are needed to assess how chatbot-generated content influences their understanding, decision-making, and satisfaction, particularly in the context of complex treatments like orthognathic treatment. This approach would provide valuable insight into the real-world utility of chatbots in supporting informed consent and shared decision-making in orthodontics.

## Conclusions

- Copilot produced the most consistent and highest-quality responses across reliability and global quality measures, whereas ChatGPT and Gemini generated outputs that were statistically more readable, although these readability differences should be interpreted cautiously given the limitations of the Flesch metric for AI-generated text.
- Copilot scored highest on modified DISCERN and GQS, with significant advantages over ChatGPT and Gemini.
- No significant differences in accuracy were observed (*P* = 0.704). Readability differed across models with ChatGPT and Gemini scoring higher than Copilot; however, all chatbot outputs fell within ‘difficult’ to ‘very difficult’ ranges requiring advanced reading ability, underscoring the need for cautious interpretation.
- Clinicians should advise patients that chatbot responses vary in accuracy, reliability, and readability. These tools may provide helpful general information, but they cannot replace the depth of expertise and personalized assessment provided by clinicians.

## Data Availability

The data underlying this article will be shared on reasonable request to the corresponding author.
